# The updating of clinical practice guidelines: insights from an international survey

**DOI:** 10.1186/1748-5908-6-107

**Published:** 2011-09-13

**Authors:** Pablo Alonso-Coello, Laura Martínez García, José Miguel Carrasco, Ivan Solà, Safia Qureshi, Jako S Burgers

**Affiliations:** 1 Iberoamerican Cochrane Centre, Institute of Biomedical Research (IIB Sant Pau), (C/Sant Antoni Maria Claret 171), Barcelona (08041), Spain; 2 CIBER of Epidemiology and Public Health (CIBERESP), Barcelona, Spain; 3 GuíaSalud-Biblioteca, Aragon Health Sciences Institute, (Avda. Gómez Laguna 25), Zaragoza, (50009), Spain; 4 Scottish National Blood Transfusion Service, (21 Ellen's Glen Road), Edinburgh, (EH17 7Q7T), UK; 5 Dutch College of General Practitioners, (Mercatorlaan 1200), Utrecht, (3528 GL), The Netherlands

## Abstract

**Background:**

Clinical practice guidelines (CPGs) have become increasingly popular, and the methodology to develop guidelines has evolved enormously. However, little attention has been given to the updating process, in contrast to the appraisal of the available literature. We conducted an international survey to identify current practices in CPG updating and explored the need to standardize and improve the methods.

**Methods:**

We developed a questionnaire (28 items) based on a review of the existing literature about guideline updating and expert comments. We carried out the survey between March and July 2009, and it was sent by email to 106 institutions: 69 members of the Guidelines International Network who declared that they developed CPGs; 30 institutions included in the U.S. National Guideline Clearinghouse database that published more than 20 CPGs; and 7 institutions selected by an expert committee.

**Results:**

Forty-four institutions answered the questionnaire (42% response rate). In the final analysis, 39 completed questionnaires were included. Thirty-six institutions (92%) reported that they update their guidelines. Thirty-one institutions (86%) have a formal procedure for updating their guidelines, and 19 (53%) have a formal procedure for deciding when a guideline becomes out of date. Institutions describe the process as moderately rigorous (36%) or acknowledge that it could certainly be more rigorous (36%). Twenty-two institutions (61%) alert guideline users on their website when a guideline is older than three to five years or when there is a risk of being outdated. Twenty-five institutions (64%) support the concept of "living guidelines," which are continuously monitored and updated. Eighteen institutions (46%) have plans to design a protocol to improve their guideline-updating process, and 21 (54%) are willing to share resources with other organizations.

**Conclusions:**

Our study is the first to describe the process of updating CPGs among prominent guideline institutions across the world, providing a comprehensive picture of guideline updating. There is an urgent need to develop rigorous international standards for this process and to minimize duplication of effort internationally.

## Background

Clinical practice guidelines (CPGs) have become increasingly popular over the last two decades. In parallel, the methodology to develop guidelines has evolved enormously [1,2]. Major attention has been given to the selection and appraisal of the available literature, becoming progressively more systematic and comprehensive. The harmonization of grading systems to classify the quality of the evidence and the strength of recommendations has been a hot issue in the guideline arena [3]. As a result, the quality of guidelines has been improved in the last decade. Nevertheless, there is still important room for improvement [4].

In guideline programs, the updating of guidelines is often scheduled irregularly [5]. Although there is no fixed lifespan for a guideline, an update every three to five years is generally recommended [6,7]. However, information about the process and methods for updating used by guideline organizations is lacking. Only few published research studies are available on this topic [6-9]. Few organizations include chapters or information on guideline updating in their handbooks on guideline development [1,2].

A significant step forward is the synthesis of available research on updating of CPGs included in the handbook of the Programme of Clinical Practice Guidelines in the Spanish National Health System. This programme is coordinated by GuíaSalud http://www.guiasalud.es, an organization created in 2002 to promote the development and use of evidence-based guidelines and other tools for improving quality of care in the Spanish Health System. Following these objectives, a common methodology for producing, implementing , and updating CPGs has been developed [10-12]. Within this context, we conducted an international survey with the aim of identifying current practices in guideline updating, exploring the need for standardization, and, ultimately, improving the guideline-updating process.

## Methods

### Design

We employed a cross-sectional design for this study.

### Study population

Our study population included key informants and experts affiliated with organizations dedicated to CPG development.

### Study sample

We selected participant institutions in spring 2009 using the following criteria: (a) members of the Guidelines International Network http://www.g-i-n.net/ that declared that they developed CPGs, (b) institutions included in the U.S. National Guideline Clearinghouse http://www.guideline.gov/ that had published more than 20 CPGs, and (c) institutions additionally selected by an expert committee based on relevance. The expert committee was composed of 12 health professionals and methodologists with experience in the field of guideline methodology and information specialists. We sent an email to each institution through the address identified via the internet. If the person receiving this email was not the person responsible for this matter, we requested that it be forwarded to whoever they considered appropriate within that institution to answer the survey.

### Intervention

We designed a self-administered survey (see Additional File 1) based on a literature review about guideline updating (unpublished). For this review, we studied websites of institutions that had published methodological handbooks and searched for published studies in MEDLINE (via PubMed) until June 2008 using a combination of descriptors (Practice Guidelines as Topic; Clinical Practice Guidelines) and free text terms (clinical guideline, practice guideline, updat*, up to date).

The survey comprised 28 items grouped into four domains. The first domain included characteristics of the organization (five items), the second was dedicated to the process of guideline updating (16 items), the third was aimed at the way users are alerted about guideline updates (two items), and the last domain focused on the future perspective on guideline updating (five items). Nineteen items included a free text area in order to gather comments or additional information.

Specific software was used to design the survey and to collect the responses http://www.surveymonkey.com. The survey was pilot tested among five institutions (three national and two international). Their feedback was used to refine the survey for optimal understanding. Between March and July 2009, we sent the survey via email to persons of selected institutions. We sent three reminders at intervals of four weeks to those institutions that had not responded. Questionnaires with no response on more than 20% of the items were returned with the request to complete the questionnaire.

### Analysis

Descriptive statistics were used to analyze the data. We calculated absolute frequencies and proportions for all items. We evaluated nonresponding institutions and compared their contact source (Guidelines International Network, National Guideline Clearinghouse, or expert committee), country, and number of CPGs produced with responding institutions using Fisher's exact test or Mann-Whitney U test (alpha was set at 0.05). We finally excluded from the analysis four items (B13-B16, Additional File 1), as they were deemed to be more related to guideline development. We assessed the guideline-updating process of responding institutions by comparing the number of years developing CPGs (≤ 10 years of experience or > 10 years of experience), contact source, and number of guidelines published per year using Fisher's exact test (alpha was set at 0.05). Data analysis was performed using SPSS statistical software, version 17.0 (SPSS Inc., Chicago, IL, USA). By consensus of the three first authors, we collected and provide the most relevant themes brought up by the responders in the free text area (responses to free text questions available from the authors on request).

Ethics approval was obtained from the hospital ethics committee (Clinical Research Ethics Committee, Hospital de la Santa Creu i Sant Pau, #74/2010).

## Results

### Characteristics of study sample

**Figure 1:**
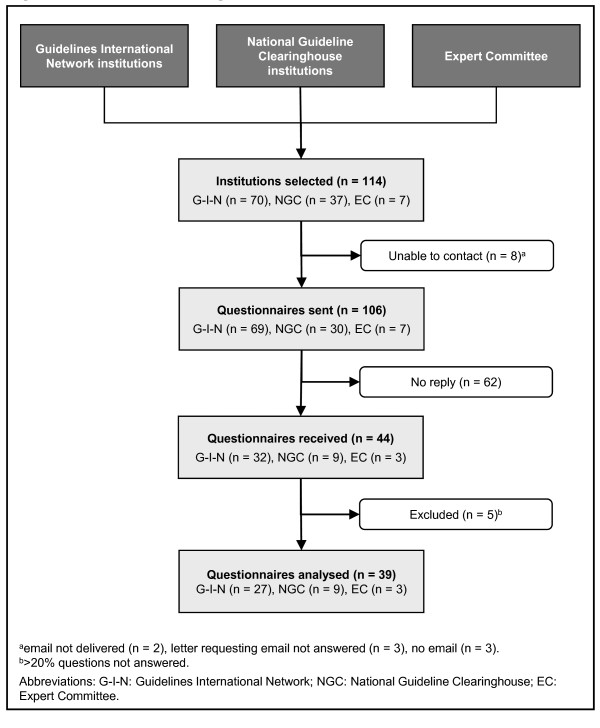
**Participation diagram**.

One hundred and fourteen institutions met at least one of the inclusion criteria. We contacted 106 of these institutions by email. We received a reply from 44 institutions (42% response rate) after three reminders. In the final analysis, we included 39 questionnaires. Five questionnaires were excluded because more than 20% of the questions were not answered (Figure 1).

Characteristics of the responding institutions are presented in Table 1. The vast majority reported that they update their guidelines (n = 36, 92%). Nonresponding and excluded institutions (n = 67) did not differ from the responding institutions with regard to their contact source (Guidelines International Network, National Guideline Clearinghouse, or expert committee; Fisher's exact test *p *= .671), country of origin (Fisher's exact test *p *= .283), and the number of guidelines produced (Mann-Whitney U test *p *= .07).

**Table 1:** Organization characteristics (n = 39)^a^

	n	(%)
**Contact source**		
Guidelines International Network	27	(69.2)
U.S. National Guideline Clearinghouse	9	(23.1)
Expert committee	3	(7.7)

**Continent**		
Europe	17	(43.6)
North America	15	(38.5)
Oceania	5	(12.8)
South America	1	(2.6)
Asia	1	(2.6)

**Type of organization**		
Scientific/professional society/association	20	(51.3)
Public institution	14	(35.9)
Other (Federal institute, nonprofit organization)	5	(12.8)

**Number of years developing guidelines**		
> 10 years	24	(61.5)
6-10 years	12	(30.8)
≤ 5 years	3	(7.7)

**Number of guidelines published^b^**		
≤ 5 per year	24	(61.5)
> 5 per year	14	(35.9)

**Updating guidelines**		
Yes	36	(92.3)
No	3	(7.7)

^a^Analysis of included institutions; ^b^One institution unknown.

### Characteristics of the guideline-updating process

**Figure 2:**
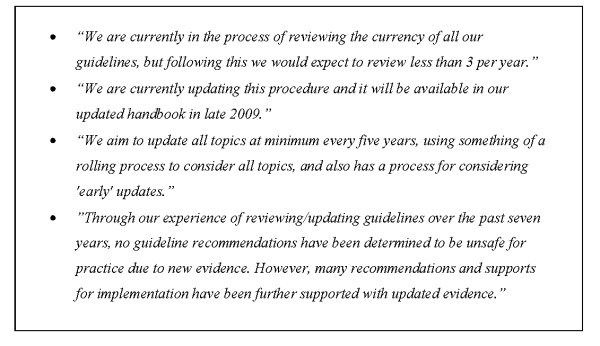
**Box of relevant comments about the characteristics of the guideline-updating process**.

Sixteen institutions (44%) reported that they check more than five guidelines for the need for annual updating, some institutions reported variable figures (n = 10, 28%), and the remaining 10 (28%) reported that they check five or less per year (Table 2, Figure 2). Over 60% of the institutions reported a time frame for considering a guideline update between three to five years. Thirty-one institutions (86%) indicated that they have a formal procedure for updating their guidelines, but only 19 (53%) have a formal procedure for deciding when a guideline becomes out of date. Nine institutions (25%) piloted the updating process to evaluate feasibility, inconveniences, or added value compared to other strategies.

**Table 2:** The guideline-updating process (n = 36)^a^

	n	(%)
**Number of guidelines checked**		
> 5 per year	16	(44.4)
Variable	10	(27.8)
3-5 per year	6	(16.7)
< 3 per year	4	(11.1)

**Number of guidelines updated**		
Unknown	14	(38.9)
≤ 5 per year	11	(30.6)
> 5 per year	7	(19.4)
Variable	4	(11.1)

**Time frame to check updating**		
3-5 years	22	(61.1)
< 3 years	11	(30.6)
Variable	3	(8.3)

**Formal procedure to update guidelines**		
Yes	31	(86.1)
No	5	(13.9)

**Formal procedure to inform about guidelines being out of date**		
Yes	19	(52.8)
No	17	(47.2)

**Formal method to decide update section or full guideline**		
No	23	(63.9)
Yes	11	(30.6)
Unknown	2	(5.6)

**Pilot testing of updating process**		
No	24	(66.7)
Yes	9	(25.0)
Unknown	3	(8.3)

**Rigor of the updating process**		
Could certainly be more rigorous	13	(36.1)
Moderately rigorous	13	(36.1)
Very rigorous	10	(27.8)

^a^Analysis of institutions updating guidelines.

**Figure 3:**
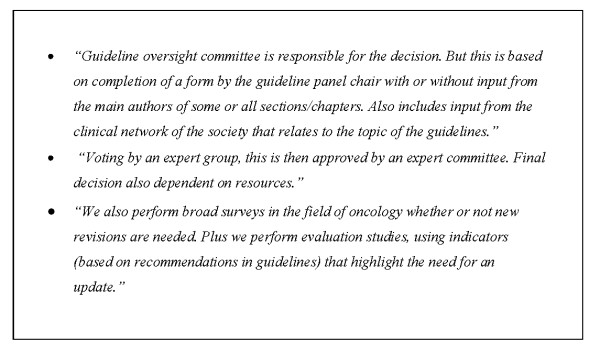
**Box of relevant comments about decision-making process of the need of updating**.

Twenty-six institutions (72%) described the process as moderately rigorous or acknowledged that it could certainly be more rigorous. Institutions that have been developing guidelines for more than 10 years are more likely to have a formal updating procedure (Fisher's exact test *p *= .047) and a rigorous process for guideline updating (Fisher's exact test *p *= .039) than are institutions who have been developing guidelines for 10 or less years (Table 3). In general, the original guideline group or an expert committee is responsible for the decision about updating the guideline (Table 4, Figure 3). The original guideline authors are most often involved in the updating process (n = 32, 89%), followed by the institution's staff (n = 30, 83%). In 13 institutions (36%), patients are involved in the process.

**Table 3:** The guideline-updating process by numbers of years developing guidelines (n = 36)^a^

	Numbers of years developing guidelines				Total		
	≤ 10 years		> 10 years				
	n	(%)	n	(%)	n	(%)	*p* ^b^
**Formal procedure to update guidelines**							
Yes	9	(69.2)	22	(95.7)	31	(86.1)	.047
No	4	(30.8)	1	(4.3)	5	(13.9)	

**Time frame to check updating**							
3-5 years	7	(53.8)	15	(65.2)	22	(61.1)	.094
< 3 years	3	(23.1)	8	(34.8)	11	(30.6)	
Varies	3	(23.1)	--		3	(8.3)	

**Rigor of the updating process**							
Could certainly be more rigorous	8	(61.5)	5	(21.7)	13	(36.1)	.039
Moderately rigorous	4	(30.8)	9	(39.1)	13	(36.1)	
Very rigorous	1	(7.7)	9	(39.1)	10	(27.8)	

^a^Analysis of institutions updating guidelines; ^b^Fisher's exact test.

**Table 4:** Characteristics of the guideline-updating process (n = 36)^a^

	Answers					
	Yes		No		Unknown	
	n	(%)	n	(%)	n	(%)
**Who decides the need for updating^b^**						
Guideline group	18	(50.0)	18	(50.0)	--	
Expert committee	15	(41.7)	21	(58.3)	--	
Guideline coordinator	9	(25.0)	27	(75.0)	--	
Other	9	(25.0)	27	(75.0)	--	
Standing editorial staff	6	(16.7)	30	(83.3)	--	

**Who participates in the updating process^c^**						
Original guideline authors	32	(88.9)	--		4	(11.1)
Staff of organization	30	(83.3)	--		6	(16.7)
New group of experts	25	(69.4)	4	(11.1)	7	(19.4)
Original information managers/specialist	21	(58.3)	5	(13.9)	10	(27.8)
Original external reviewers	20	(55.6)	6	(16.7)	10	(27.8)
Patients	13	(36.1)	11	(30.6)	12	(33.3)
Others	7	(19.4)	5	(13.9)	24	(66.7)

**Which part of the guidelines get checked^c^**						
Full text	29	(80.6)	2	(5.6)	5	(13.9)
All recommendations	29	(80.6)	1	(2.8)	6	(16.7)
Key questions	25	(69.4)	1	(2.8)	10	(27.8)
Key recommendations	25	(69.4)	--		11	(30.6)
Annexes	20	(55.6)	3	(8.3)	13	(36.1)
Patient information	19	(52.8)	5	(13.9)	12	(33.3)

**Which kind of search run^b^**						
Original search strategies plus some horizon scanning	20	(55.6)	16	(44.4)	--	
Original searches strategies modified to be specific rather than sensitive	14	(38.9)	22	(61.1)	--	
Original search strategies	10	(27.8)	26	(72.2)	--	
Other	7	(19.4)	29	(80.6)	--	

^a^Analysis of institutions updating guidelines; ^b^Closed-ended questions yes/no; ^c^Aggregation responses yes/partially.

**Figure 4:**
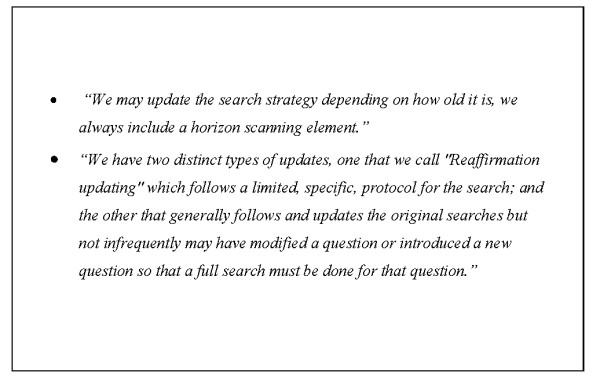
**Box of relevant comments about the characteristics of the search process**.

Institutions tend to check and review different parts of the guideline when deciding about the need to update a guideline. Twenty-nine institutions (81%) said they check all recommendations and the full guideline text. Less frequently, key questions and recommendations, supplementary annexes, and patient information are checked. The institutions use several search strategies (Table 4, Figure 4). Twenty institutions (56%) ran the original search strategies and did additional horizon scanning, 14 institutions (40%) use more specific strategies than the original strategies, and seven (20%) institutions run other searches. Twenty-two institutions (61%) alert guideline users on their website when a guideline is older than three to five years or when there is a risk of being outdated.

### Future plans for updating guidelines

**Figure 5:**
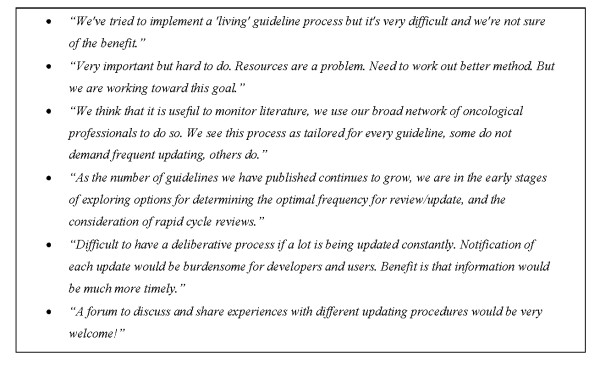
**Box of relevant comments about future plans for updating guidelines**.

Twenty-five institutions (64%) supported the concept of "living guidelines" (Table 5, Figure 5), defined as guidelines that are continuously monitored and updated [13]. The majority of institutions, however, reported difficulties and inconvenience in putting this concept in practice. Almost half of the institutions reported that they have plans to improve their guideline-updating process (n = 18, 46%). More than half of the institutions are willing to share resources with other organizations (n = 21, 54%). However, only 20% of the organizations reported that they would rely on other guidelines when updating or developing a guideline.

**Table 5:** The guideline-updating process in the future (n = 39)^a^

	Answers					
	Yes		No		Not sure/unknown	
	n	(%)	n	(%)	N	(%)
**It is worth having living guidelines^b^**	25	(64.1)	6	(15.4)	8	(20.5)

**Plans to set up a protocol to improve the updating process**	18	(46.2)	10	(25.6)	11	(28.2)

**Share resources with other organizations**	21	(53.8)	1	(2.6)	17	(43.6)
**Resources to share (n = 21)**						
- References	20	(95.2)	--		1	(4.8)
- Evidence synthesis	19	(90.5)	--		2	(9.5)
- Key questions	18	(85.7)	--		3	(14.3)
- Search strategies	18	(85.7)	--		3	(14.3)
- Evidence tables	18	(85.7)	1	(4.8)	2	(9.5)
- Considered judgement forms^c^	14	(66.7)	--		7	(33.3)

^a^Analysis of included institutions; ^b^Considering "living guidelines" as those that are continuously being monitored and updated; ^c^Document that explicitly includes the factors taken into account when grading recommendations.

## Discussion

Our study is the first international survey about the process of updating CPGs among guideline institutions across the world. Although most institutions reported having a process for updating guidelines, the process is not standardized and could be more rigorous. Many guideline developers, including those with long-standing experience, reported that they have plans to improve this process. Others are waiting for more evidence before modifying their current system.

Surprisingly, half of the organizations do not have a formal process for deciding when a guideline becomes outdated. Guideline developers need to recognize this limitation when promoting guidelines as support tools for the practice of evidence-based medicine. Similarly, guideline users should be cautious when relying on guidelines of a certain age. This lack of rigor in methodology in general was recently found in a systematic review about the quality of guidelines in the last two decades [4,14]. On the other hand, most organizations in our survey showed awareness about using insufficient methods for updating guidelines and intended to improve their processes. Up to 72% think that their updating process is only moderately rigorous or could be more rigorous. This is an issue that guideline developers need to address. This finding is consistent with the fact that only 20% of organizations in our survey would rely on other guidelines when updating or developing a guideline. This is an unfortunate paradox given the actual scenario, where most institutions would like to be able to share the burden of the development process. There is a perceived need for international collaboration, but the product to be exchanged needs to be more mature.

The majority of institutions support the concept of living guidelines. However, this type of guideline development is regarded as very labour intensive and resources may be insufficient. This modality could make more sense in fast-changing fields such as AIDS, cardiovascular risk management, and breast cancer. Guidelines on other topics, such as venous ulcer or sinusitis, may need less frequent updating. Some responders emphasized that guideline updating should be tailored to the topic in order to optimize the efficient use of resources (Figure 5).

A noted limitation of frequent updating of guidelines is that notifications of each update could be burdensome for developers and users (Figure 5). Users' interests may vary for different kinds of updates, some being interested in any change made to the guideline, some just being concerned about major modifications. Ideally, web-based organizations could have personalized systems of alerts that could be tailored to each user group.

Sufficient funding is important for appropriate guideline updating. Guideline organizations that are structurally embedded within the countries' healthcare system and funded by the government, such as the National Institute for Health and Clinical Excellence (NICE) and the Scottish Intercollegiate Guidelines Network (SIGN), have more rigorous updating procedures. In organizations with fewer resources, funding is only available for developing *de novo *guidelines. Research in the field of guideline updating is scarce. There is an urgent need for valid tools to estimate the rate of new relevant findings related to the topic of the guideline and for efficient search strategies to track new research evidence. In addition, more knowledge is needed about the best method to reach end users when guidelines are out of date and when guidelines are updated.

Our survey shows that institutions consider guideline updating to be time consuming and resource intensive. Despite the limitations described above, over half of the institutions surveyed are eager to share the burden and work with peer institutions. International collaboration could further help to avoid duplication of effort. Some institutions suggested that a forum to discuss and share updating experiences would be helpful (Figure 5). The Guidelines International Network could provide these facilities, in the same way that they support other groups active in guideline methodology.

Work is being duplicated around the world, with institutions failing to work jointly, consolidating networks around health topics or fields. Timidly but progressively, international collaboration on guideline development and updating for chronic obstructive pulmonary disease (COPD) has been initiated recently [15]. In the field of oncology, a European collaboration of guideline institutions (CoCanCPG) has been active [16]. To increase the efficient use of existing guidelines in guideline updating, the ADAPTE methodology could be helpful [17]. In addition, a standardized format for evidence tables and for grading the evidence could help with sharing evidence worldwide [3,18]. Finally, international databases of gaps in evidence could be developed, which could feed the agenda of healthcare researchers and reviewers, such as the Cochrane Collaboration.

This study has a few limitations. First, the response rate was rather low, despite sending three reminders. Nevertheless, our survey included the most prominent guideline organizations, like NICE, SIGN, the United States Preventive Services Task Force, and the New Zealand Guidelines Group (Additional File 2). We did not find essential differences between responding and nonresponding institutions. Second, bias cannot be excluded due to the nature of the survey being self-reported. Although we contacted a key informant from each institution, other responders from the same institutions might have provided different answers. In some institutions, the person initially contacted referred us to another person more able to answer the questions, which increases the likelihood of appropriate answers.

## Conclusions

Our study provides the first comprehensive picture of guideline updating around the world. This stage in guideline development has not benefited from the same rigor of methodological development that has been applied to the initial development of a guideline. Our study shows that it is an area that needs increasing attention. Our main findings include the urgent need to develop a rigorous standard for this process, initially by funding research into how to optimize the process, share the burden, and minimize duplication of effort internationally. We believe that these changes will improve the quality and impact of guidelines and, ultimately, patient care.

## Competing interests

The authors declare that they have no competing interests.

## Authors' contributions

PAC, LMG, JMC, IS, SQ, and JSB participated in the conception and design of the study. LMG, PAC, and JMCG analyzed the data. PAC and LMG drafted a first version. All members of the Updating Guidelines Working Group participated in the design of the study and revising the draft critically for important intellectual content and all authors have given final approval of the version to be published.

## Supplementary Material

Additional file 1 **Survey**. This document shows the survey designed, based on a literature review about guideline updating. Click here for file

Additional file 2 **Organizations**. This document shows information about the organizations that participated in this survey (name, country and source of contact). Click here for file
