# Supplementary material for: The updating of clinical practice guidelines: insights from an international survey
Source: Implement Sci. 2011 Sep 13;6:107. doi: 10.1186/1748-5908-6-107 (PMC3191352; doi:10.1186/1748-5908-6-107)
Supplement: Additional file 1 — Survey. This document shows the survey designed, based on a literature review about guideline updating. [file 1748-5908-6-107-S1.PDF]

Additional file 1

Survey

A. Organization characteristics

A1. What is the name of your organization?

A2. Which kind of organization is your institution?
- ☐ Public institution
- ☐ Private organism
- ☐ Scientific society
- ☐ Other

If other, please specify……..

A3. How many years have you been developing guidelines?
- ☐ Less than 3 years
- ☐ 3-5 years
- ☐ 6-10 years
- ☐ More than 10 years

A4. How many guidelines do you publish per year?
- ☐ Less than 3 per year
- ☐ 3-5 per year
- ☐ 6-10 per year
- ☐ More than 10 per year

A5. Does your organization update its guidelines?
- ☐ Yes
- ☐ No

B. The updating process

B1. How many guidelines per year do you check to asses the need of updating?
- ☐ Less than 3
- ☐ 3-5 per year
- ☐ 6-10 per year
- ☐ More than 10 per year
- ☐ Varies.

If varies, please specify……

B2. How many do you actually update (partially or globally)?
- ☐ None
- ☐ Less than 3
- ☐ 3-5 per year
- ☐ 6-10 per year
- ☐ More than 10
- ☐ Varies

If you know the actual percentage of Guidelines do you update, please specify………………………………………...

B3. Does your organization have a formal procedure to update your guidelines or recommendations?
- ☐ No
- ☐ Yes

If YES please specify if it is published somewhere and if available on-line provide the link…………………...............

B4. Do you have a specific time frame to decide when to check for the need of updating a guideline?
- ☐ No, it varies
- ☐ Less than 3 years
- ☐ 3 years
- ☐ 4-5 years
- ☐ More than 5 years

B5. Who decides the need for updating? (More than one option possible)

Guideline coordinator	☐ No	☐ Yes
Guideline group	☐ No	☐ Yes
Expert committee	☐ No	☐ Yes
Standing Editorial staff	☐ No	☐ Yes
Other	☐ No	☐ Yes

If guideline group please specify if the original group or just a few. If other please specify…………………………….

B6. Which parts of the guideline do you check?

Key recommendations	☐ No	☐ Yes	☐ Partially

All recommendations	☐ No	☐ Yes	☐ Partially
Key questions	☐ No	☐ Yes	☐ Partially
Full text	☐ No	☐ Yes	☐ Partially
Patient information	☐ No	☐ Yes	☐ Partially
Annexes	☐ No	☐ Yes	☐ Partially

B7. Do you have a formal process to decide when a guideline becomes out of date (e.g. if new evidence available)?

☐ No
☐ Yes

If yes, please specify……………………………………………………………………………………………………….

B8. Do you have a formal method to reach a consensus or to decide whether to update just a section or the full guideline?

☐ No
☐ Yes

If yes please specify (Delphi, voting, etc.)……………………………………………………………………………….

B9. Who participates in the updating process?

Original guideline authors	☐ No	☐ Yes	☐ Sometimes
Original information managers/specialist	☐ No	☐ Yes	☐ Sometimes
Original external-reviewers	☐ No	☐ Yes	☐ Sometimes
New group of experts	☐ No	☐ Yes	☐ Sometimes
Patients	☐ No	☐ Yes	☐ Sometimes
Staff of organization	☐ No	☐ Yes	☐ Sometimes
Others	☐ No	☐ Yes	☐ Sometimes

If others or sometimes please specify…………………………………………………………………………………

B10. What kind of searches do you run when updating?

Original search strategies	☐ No	☐ Yes
Original searches strategies modified to be specific rather than sensitive	☐ No	☐ Yes
Original search strategies plus some horizon scaning (eg. new treatments or technology)	☐ No	☐ Yes
Other	☐ No	☐ Yes

Please, comment your answer……………………………………………………………………………………………

B11. Has the updating process used in your organization been piloted?

☐ No
☐ Yes

If yes, please specify………………………………………………………………………………………………………

B12. How rigorous do you think is the updating process that you use?

☐ Unreliable
☐ Not very reliable
☐ Could certainly be more rigorous
☐ Moderately rigorous
☐ Very rigorous

B13. Do you check for other guidelines about the same topic of interest when developing a guideline?[a]

☐ No
☐ Yes

B14. In case you do, do you trust their evidence synthesis and update only from the search date of this guideline?[a]

☐ Yes
☐ No, we only use them to check if we miss relevant references
☐ No, never
☐ Others

If others, please specify……………………………………………………………………………………………………

B15. Does your organization adapt CPG when developing your own?[a]

☐ No
☐ Yes

B16. In case you do, does your organization take part in the ADAPTE Collaboration?[a]

☐ No
☐ Yes

<u>C. Updating and users</u>

C1. Do you alert guideline users on your website when a guideline is older than a certain number of years or when there is a risk of being outdated?

☐ No
☐ Yes

If yes please specify how you do it……………………………………………………………………………………

C2. How do you alert guideline users when a modification has taken place?

D. Updating CPG in the future

D1. Do you think is it worth having living guidelines? Considering "living" as guidelines that are continuously being monitored and updated.

- ☐ No
- ☐ Yes
- ☐ Not sure

Regardless of your answer please comment pros and cons……………………………………………………………….

D2. Do you have plans in your organization to set up a protocol to improve the updating of your guidelines?

- ☐ No
- ☐ Yes
- ☐ Maybe

If yes, please specify……………………………………………………………………………………………………

D3. Would your organization be willing to share resources to optimize guideline updating and/or developing process internationally?

- ☐ No
- ☐ Yes
- ☐ Not sure

If not sure please tell us why or what would it depend on…………………………………………………………………

D4. Which resources would you be willing to share with other organizations?

Key questions	☐ No	☐ Yes	☐ Not sure	☐ Not worth it
Search strategies	☐ No	☐ Yes	☐ Not sure	☐ Not worth it
References	☐ No	☐ Yes	☐ Not sure	☐ Not worth it
Evidence tables	☐ No	☐ Yes	☐ Not sure	☐ Not worth it
Evidence synthesis	☐ No	☐ Yes	☐ Not sure	☐ Not worth it
Considered judgement forms (document that explicitly includes the factors taken into account when grading recommendations)	☐ No	☐ Yes	☐ Not sure	☐ Not worth it

E. Please comment about any issues you find relevant, and you want to mention, regarding updating clinical guidelines that might be of use for our survey. Thank you for your help!

[a]Excluded item
