# Supplementary material for: The updating of clinical practice guidelines: insights from an international survey
Source: Implement Sci. 2011 Sep 13;6:107. doi: 10.1186/1748-5908-6-107 (PMC3191352; doi:10.1186/1748-5908-6-107)
Supplement: Additional file 2 — Organizations. This document shows information about the organizations that participated in this survey (name, country and source of contact). [file 1748-5908-6-107-S2.PDF]

Additional file 2

Included institutions (alphabetical classification by name organization)

	Name of organizations	Countries	Source of contact
1	Agency for Quality in Medicine	Germany	G-I-N
2	American College of Physicians	USA	G-I-N
3	American Urological Association	USA	G-I-N
4	American Academy of Otolaryngology	USA	G-I-N
5	American Academy of Paediatrics	USA	NGC
6	American College of Cardiology	USA	Expert Committee
7	American College of Chest Physicians	USA	G-I-N
8	American College of Obstetricians and Gynaecologists	USA	NGC
9	American College of Radiology	USA	NGC
10	Basque Office for Health Technology Assessment	Spain	G-I-N
11	Belgian Health Care Knowledge Centre	Belgium	Expert Committee
12	Brazilian Medical Association	Brazil	G-I-N
13	British Columbia Council on Clinical Practice Guidelines	Canada	NGC
14	CARI Guidelines	Australia	G-I-N
15	Catalan Agency for Health Technology Assessment and Research	Spain	G-I-N
16	Current Care / Duodecim - Finnish Medical Society	Finland	G-I-N
17	Domus Medica vzw; Flemish College of General Practitioners	Belgium	G-I-N
18	Duodecim Medical Publications Ltd	Finland	G-I-N
19	Dutch Association of Comprehensive Cancer Centres	Netherlands	G-I-N
20	Dutch Institute for Healthcare Improvement	Netherlands	G-I-N
21	German Cancer Society	Germany	G-I-N
22	Guidelines Advisory Committee	Canada	NGC
23	Health Austria, Federal Institute for Quality in Health Care	Austria	G-I-N
24	HTA Unit, Ministry of Health, Malaysia	Malaysia	G-I-N
25	Hungarian Ministry of Health	Hungary	Expert Committee
26	Infectious Diseases Society of America	USA	G-I-N
27	Italian National Institute of Health	Italy	G-I-N
28	Joanna Briggs Institute	Australia	G-I-N
29	Kidney Disease Improving Global Outcomes	USA	NGC
30	Michigan Quality Improvement Consortium	USA	NGC
31	National Heart Foundation of Australia	Australia	G-I-N
32	National Institute for Clinical Excellence	United Kingdom	G-I-N
33	New Zealand Accident Compensation Corporation	New Zealand	G-I-N
34	New Zealand Guidelines Group	New Zealand	G-I-N
35	Registered Nurses Association of Ontario	Canada	NGC
36	Royal Dutch Society for Physical Therapy	Netherlands	G-I-N
37	Scottish Intercollegiate Guidelines Network	United Kingdom	G-I-N
38	Trimbos Institute Netherlands Institute of Mental Health & Addiction	Netherlands	G-I-N
39	United States Preventive Services Task Force	USA	NGC

Abbreviations: G-I-N: Guidelines International Network; NGC: National Guideline Clearinghouse
